# Thermal stability of hyper-doped n-type Ge and Si_0.15_Ge_0.85_ epilayers obtained by *in situ* doping and pulsed laser melting

**DOI:** 10.1039/d5tc02390d

**Published:** 2025-07-21

**Authors:** Marco Faverzani, Giulia Maria Spataro, Davide Impelluso, Stefano Calcaterra, Enrico Di Russo, Michele Magnozzi, Francesco Bisio, Maurizio Canepa, Paolo Biagioni, Giovanni Isella, Enrico Napolitani, Jacopo Frigerio

**Affiliations:** a Dipartimento di Fisica, Politecnico di Milano piazza L. da Vinci 32 20133 Milano Italy marco.faverzani@polimi.it; b Dipartimento di Fisica e Astronomia, Università di Padova, via Marzolo 8 35131 Padova Italy; c Istituto Nazionale di Fisica Nucleare, Laboratori Nazionali di Legnaro, viale Dell’Università 2 35020 Legnaro (PD) Italy; d OptMatLab, Dipartimento di Fisica, Università di Genova, via Dodecaneso 33 16146 Genova Italy; e CNR-SPIN, corso Perrone 24 16152 Genova Italy

## Abstract

The thermal stability of hyper-doped Ge-on-Si and SiGe-on-Si films featuring carrier concentrations exceeding 5 × 10^19^ cm^−3^ obtained by *in situ* doping and pulsed laser melting has been studied. The deactivation kinetics was systematically analysed through rapid thermal annealing, reflection spectroscopy and electrical characterization. The results demonstrate that, while hyper-doped Ge films exhibit rapid deactivation at temperatures above 300 °C, SiGe offers enhanced thermal stability. Surface morphology analysis confirms the preservation of flatness after pulsed laser melting and thermal treatments, suggesting possible exploitation of these materials as substrates for further growth. These findings provide insights into optimizing hyper-doped material platforms for mid-infrared photonic devices and advanced semiconductor applications, emphasizing the trade-offs between the initial carrier concentration and the thermal resilience.

## Abstract 2

Pulsed laser melting enables high electron activation in Ge and SiGe epilayers with the latter exhibiting enhanced thermal stability. Our findings highlight the trade-off between the activation level and dopant resilience.

## Graphical abstract

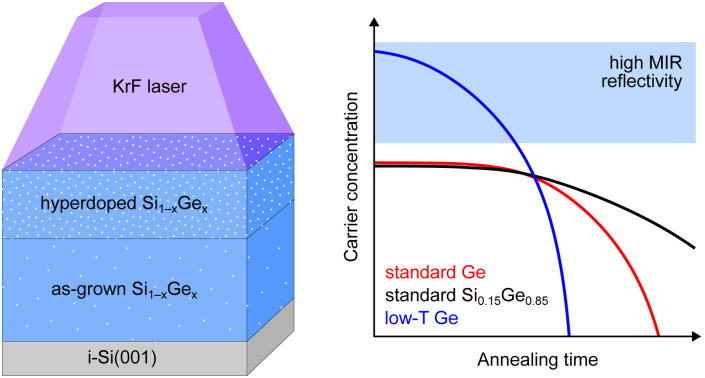

## Introduction

In the last 20 years germanium has drawn a lot of attention for its remarkable electrical and optical properties as well as for its compatibility with mature silicon technology. Indeed, germanium shows higher carrier mobilities than silicon for both electrons and holes making it an appealing material for the realization of high-mobility channel MOSFETs,^1^ which however require high n- and p-type doping levels and low-resistivity ohmic contacts. Furthermore, due to its quasi-direct band gap, many efforts have been devoted to the implementation of a Ge-on-Si laser obtained by band structure engineering through strain, doping and alloying with tin.^2–4^ Besides, germanium is one of the most promising materials for the realization of mid-infrared (MIR) photonic integrated circuits thanks to its wide transparency window, ranging from 3 to 14 μm, which covers the technologically relevant fingerprint region.^5^ Similarly, silicon–germanium alloys have been employed for the realization of MIR waveguides exploiting the flexibility of the alloy composition for the control of the refractive index profile.^6,7^ Germanium has also been considered as a CMOS-compatible alternative to the conventional plasmonic materials as its plasma frequency can be tuned by acting on the doping level and this ultimately led to the implementation of germanium antennas directly grown on silicon for plasmon-enhanced sensing.^8–10^ Since doped semiconductors may serve as mirrors in the MIR spectral region, highly-reflective germanium or silicon–germanium films could be employed, for instance, as reflectors for the realization of photonic microcavities which are needed for the investigation of the intersubband strong coupling regime in group-IV-based multiple quantum wells.^11^ Such microcavities are usually defined by two metallic mirrors enclosing the heterostructure, a geometry which requires the removal of the substrate during the fabrication for the integration of the bottom mirror. This could be possibly avoided by replacing the bottom metallic mirror with a doped semiconductor, thus allowing for the monolithic growth of the whole system. In all these applications, doping plays a significant role and to take full advantage of the superior optoelectronic properties of germanium, a reliable way of achieving high doping levels is required. While the realization of hyper-doped p-Ge is nowadays well-established,^12–16^ stable n-Ge featuring an electron density exceeding 5 × 10^19^ cm^−3^ remains challenging. In this context, it is important to highlight that, whereas hyper-doping in Si is achieved when exceeding the dopant solid solubility limit, in Ge it is attained by reaching the maximum equilibrium active concentration, *i.e.* around 6 × 10^19^ cm^−3^ for phosphorous-doped Ge.^17^

In germanium epitaxial films grown on silicon substrates at temperatures around 500 °C employing GeH_4_ as precursor gas and *in situ* doping with PH_3_ during the growth, the maximum attainable carrier density is usually limited to values around 2 × 10^19^ cm^−3^ because of the unavoidable formation of neutral vacancy-dopant complexes. Prucnal *et al.*^18^ theoretically calculated the equilibrium concentration of substitutional P atoms, as well as that of P_*n*_V_*m*_ complexes in germanium, considering a total phosphorous density of 10^20^ cm^−3^, close to the equilibrium solid solubility limit of phosphorous in germanium,^19^ finding that, at temperatures lower than 700 °C, substitutional-P and P_4_V defects prevail and show similar concentrations, thus suggesting that the percentage of activated dopants cannot exceed 20%. This means that at equilibrium many dopants do get incorporated but only a few of them are electrically active.

A viable way of further pushing this limit is offered by post-growth out-of-equilibrium processes such as flash lamp annealing (FLA) and pulsed laser melting (PLM). Indeed, although post-growth rapid thermal annealing (RTA) can be performed to slightly increase the activation, it is plagued by the transient enhanced diffusion typically occurring in all thermal processes lasting more than a few seconds. Hence, to avoid this drawback, annealing times well below one second are needed^20^ which can be achieved in FLA (ms range) and PLM (tens of ns range). The latter, specifically, firstly dissolves the complexes and then induces a rapid liquid-phase epitaxial regrowth during which the diffusion is limited to the molten region, therefore favouring the uniform distribution of the dopants and consequently their electrical activation. Moreover, the thickness of the molten region can be controlled by carefully choosing the energy density of the employed laser.^17^ Controlling the thickness of the hyper-doped layers is particularly useful when dealing with MIR mirrors since, in this case, having a high doping level is not enough if the carriers are distributed over a relatively thin region whose thickness is lower than the skin-depth.

Some of the aforementioned applications also require post-growth processes carried out at moderate-to-high temperatures, such as the formation of good electrical contacts exhibiting ohmic behaviour on germanium, which often involves annealing treatments at temperatures between 300 and 600 °C.^21^ Besides, to fully exploit the potential of hyper-doped germanium films, using them as substrates for further epitaxial growth could offer novel possibilities. Carrying out the growth without deactivating all the dopants, however, requires a careful choice of the thermal budget, and therefore knowledge of the thermal stability of the carrier concentration in the hyper-doped films. Hence, in this article we experimentally investigate the thermal stability of Ge-on-Si and Si_0.15_Ge_0.85_-on-Si films obtained by *in situ* doping and subsequent PLM, performing a systematic study of the deactivation kinetics as a function of the annealing conditions.

## Materials and methods

The samples were grown by low-energy plasma-enhanced chemical vapour deposition (LEPECVD)^22^ on 100 mm Si(001) substrates and *in situ* doped by fluxing PH_3_ into the chamber during the growth. The standard germanium film, hereafter labelled as GeA, features a 2 μm-thick Ge epilayer grown on a single-side-polished intrinsic Si(001) substrate with resistivity larger than 6000 Ω cm. The deposition has been carried out at the standard temperature of 500 °C with a growth rate of 1.15 nm s^−1^ and using 20 sccm of GeH_4_ and 0.35 sccm of PH_3_. The SiGe sample is constituted by a 2 μm-thick epilayer of Si_0.15_Ge_0.85_ grown on a single-side-polished intrinsic Si(001) substrate with resistivity larger than 6000 Ω cm. The epitaxial growth has been carried out at the standard temperature of 550 °C with a rate of 1.39 nm s^−1^ and using 20 sccm of GeH_4_, 3.75 sccm of SiH_4_ and 0.35 sccm of PH_3_. A second germanium sample, hereafter labelled as GeB, has been grown at lower temperature and growth rate to increase the incorporation of the phosphorus atoms^23–25^ while maintaining good crystal quality. GeB features a 500 nm-thick Ge epilayer grown on a double-side-polished intrinsic Si(001) substrate with resistivity larger than 10 000 Ω cm. The deposition has been carried out at 350 °C with a growth rate of 0.03 nm s^−1^ and using 0.3 sccm of GeH_4_ and 0.05 sccm of PH_3_.

The PLM processes were carried out with a Coherent COMPex 201 KrF excimer laser (248 nm wavelength and 22 ns pulses) equipped with a beam homogenizer allowing for the uniform illumination of an area of 5 × 5 mm^2^. GeA and GeB were treated with a single pulse having an energy density of 900 mJ cm^−2^ which was reduced to 500 mJ cm^−2^ for the SiGe samples.

The phosphorous content profiles were characterized by secondary ion mass spectrometry (SIMS) with a Cameca IMS-4f instrument. A 5.5 keV Cs^+^ primary beam was rastered over a 250 × 250 μm^2^ square spot while collecting ^133^Cs^31^P^+^ secondary ions. The calibration of the phosphorous concentration was performed measuring a Ge standard of known P density, with an accuracy of 10%, while the depth scale was calibrated by measuring the crater depths with a Tencor P-17 profilometer and assuming a constant sputtering rate, with an accuracy of 2%.

The carrier concentration was deduced from the near-normal incidence, *i.e.* at around 10° incidence angle, reflectivity spectra acquired between 400 (25) and 5000 (2) cm^−1^ (μm) with a Bruker Invenio Fourier transform infrared (FTIR) spectrometer equipped with a globar source, a KBr beam splitter and a deuterated alanine-doped tri-glycine sulphate pyroelectric photodetector. The beam impinging onto the sample covers an area of approximately 1 × 1 mm^2^. The spectra were fitted according to a multilayer model applying the transfer matrix method: a Drude-like dielectric function was employed to describe the optical response of the doped Ge and SiGe layers while the tabulated optical constants of silicon^26^ were considered to describe the substrate. The free-carrier concentration *n* was then extracted from the plasma frequency by applying the relation1
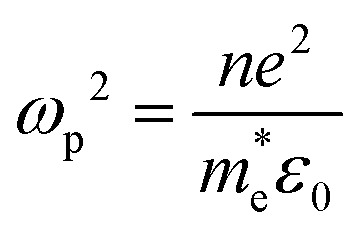
with 
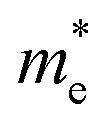
 being the conductivity effective mass of the electrons.

Additionally, variable-angle ellipsometric measurements were performed to further validate the adopted model employing a SENDIRA infrared spectroscopic ellipsometer (IRSE) by SENTECH Instruments GmbH on selected samples over the spectral region extending from 500 (20) to 6000 (1.67) cm^−1^ (μm) and at incidence angles of 55°, 60° and 65°.

For comparison, the carrier concentration was also determined by Hall effect measurements acquired in the Van der Pauw (VdP) configuration with a four-point probe apparatus.^27^ Also in this case, a multilayer model was assumed for the analysis of the experimental data within the integral approach.^28,29^ Assuming uniform properties within each layer, the measured sheet resistivity *R*_s_ and Hall coefficient RH_s_ can be related to those of the three layers, *i.e.* the Si substrate, the epitaxial layer and the hyper-doped layer. Since the substrate does not significantly contribute to the overall electrical response due to its low doping level, measurements on as-grown samples readily yield the properties of the epitaxial layers which can then be employed to retrieve the properties of the hyper-doped layer from measurements performed on PLM-treated samples. Once the sheet resistivity and the Hall coefficient of each layer were determined, the Hall dose *n*_H_ can be calculated according to *n*_H_ = *γ*_H_/(*e*·RH_s_) where *γ*_H_ is the Hall scattering factor whose experimental value is around 0.9 for both germanium^30^ and silicon,^31^ and is therefore expected to be approximately the same also for silicon–germanium alloys. The depth profile of the electron concentration was measured by a differential VdP–Hall technique alternating four-point probe measurements and chemical etching with an aqueous hydrogen peroxide solution with 3% wt H_2_O_2_ for the layer removal.^27^ The depth scale was determined by measuring the removed thickness after each step with a profilometer, with an estimated accuracy of about 10%.

To investigate the thermal stability of the hyper-doped films, RTA thermal treatments were performed at different temperatures and different annealing durations. The samples characterized by FTIR spectroscopy were annealed in a UniTemp RTP-150-HV oven under vacuum conditions: after having reached a pressure below 10^−5^ mbar, the sample is firstly heated with a ramp-up of 10 °C s^−1^ up to the annealing temperature which is then maintained for the duration of the thermal process; finally, the sample is rapidly cooled down with a N_2_ flux of 5 L min^−1^. The samples characterized by electrical VdP–Hall measurements were instead processed with an RTA Jipelec JetFirst-150 in a N_2_ atmosphere. Though the nominal annealing conditions were the same, the temperature was monitored during the processes with a class-2 type-K thermocouple soldered to the Si wafer acting as a holder for the samples.

The surface morphology was studied by atomic force microscopy (AFM) with a commercial Bruker Innova microscope covering a 10 × 10 μm^2^ area in tapping mode. AFM maps were post-processed applying polynomial background subtraction and scan line shift correction.

## Results and discussion

### Chemical profile and electrical characterization

**Fig. 1:**
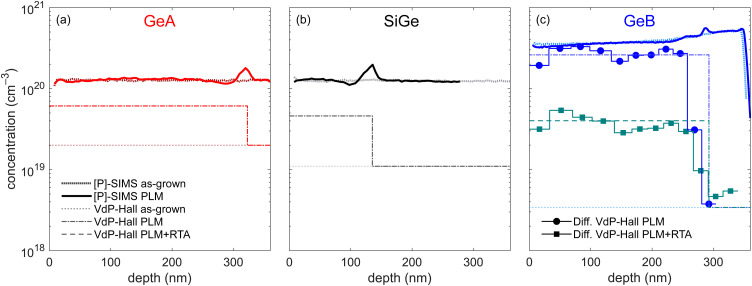
Incorporated and active carrier density retrieved by electrical VdP–Hall measurements before and after PLM process for (a) GeA, (b) SiGe and (c) GeB. The depth profiles of the active carrier concentration after PLM and after PLM and RTA for GeB are also shown.

For all samples, the incorporated P concentration and the active carrier density have been determined by SIMS and VdP–Hall measurements, respectively. As shown in Fig. 1(a), as-grown GeA features a flat chemical profile with a P concentration of 1.25 × 10^20^ cm^−3^, which remains almost unaltered after the PLM process, apart from the appearance of an accumulation peak located at a depth of about 320 nm, corresponding to the maximum melt depth (MMD), which is due to the P pile-up occurring at the interface between the molten region and the underlying material.^32^ The as-grown sample features an active carrier concentration of 2 × 10^19^ cm^−3^, which increases up to 6.1 × 10^19^ cm^−3^ in the molten region after PLM. The SiGe sample shows a similar behaviour, featuring a P chemical concentration of about 1.25 × 10^20^ cm^−3^ both before and after the PLM process.

In this case, however, laser annealing with an energy density of 900 mJ cm^−2^ causes surface cracks which could be detrimental for subsequent processing steps and the employed energy density was hence reduced to 500 mJ cm^−2^, resulting in the reduction of the maximum melt depth so that the accumulation peak is located much closer to the surface, at a depth of approximately 135 nm. Also in this case, the carrier concentration significantly increases after the PLM process, from 1 × 10^19^ to 4.6 × 10^19^ cm^−3^. GeB exhibits a significantly different behaviour: indeed, the low-temperature growth protocol employed for this sample resulted in a remarkable increase in the P concentration, which exceeds 3.5 × 10^20^ cm^−3^ in the as-grown sample and remains almost unchanged after the PLM process, with an accumulation peak at the MMD, located at a depth of 280 nm. It is worth noting that, despite the very high chemical concentration of P atoms, the active carrier concentration of the as-grown sample is limited to around 3.5 × 10^18^ cm^−3^ with this low active carrier density tentatively being attributed to the increased vacancy concentration in the epilayer, which results from the low-temperature epitaxial growth and leads to a higher fraction of inactive dopants. To gain further insights into the vertical distribution of the carriers after the PLM and RTA processes, the differential VdP–Hall characterization technique was employed for GeB. In Fig. 1(c) it can be noticed that, after PLM, the active carrier concentration leaps to 2.6 × 10^20^ cm^−3^, thanks to the dissolution of vacancy-dopant complexes provided by PLM. The vertical profile of the active carrier density remains relatively flat, up to a depth approaching the MMD, at which it sharply drops, eventually reaching the carrier density of the as-grown sample. To determine whether this uniformity is preserved even after an RTA process, further differential VdP–Hall characterization studies were performed after annealing the samples at gradually increasing temperature up to 325 °C, as discussed in the section focusing on isochronal thermal treatments, finding an average carrier density in the hyper-doped region which drops to 4 × 10^19^ cm^−3^, while maintaining also in this case a relatively flat profile up to the MMD.

### Morphological characterization

**Fig. 2:**
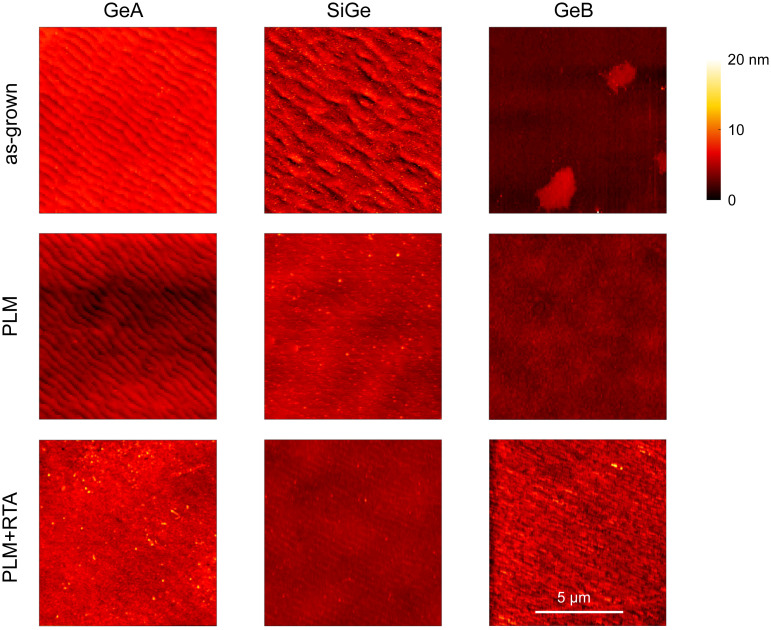
10 × 10 μm^2^ AFM maps of the samples after the epitaxial growth, after the PLM process and after the isochronal thermal treatments discussed in the text.

Fig. 2 shows the AFM maps of the investigated samples to illustrate the surface morphology of the as-grown samples, the PLM-processed samples and, eventually, the samples after several thermal treatments, *i.e.* the isochronal treatments discussed later in the text. The root-mean-square (RMS) roughness extracted from the AFM images is summarized in Table 1.

**Table 1:** RMS surface roughness retrieved from the AFM maps shown in Fig. 2

Sample	RMS roughness [nm]		
	As-grown	PLM	PLM + RTA
GeA	2.0	1.0	1.2
SiGe	1.1	0.9	0.5
GeB	0.9	0.5	1.7

The as-grown GeA sample exhibits periodic ripples with a periodicity of approximately 0.5 μm, while maintaining an overall flat surface with a RMS roughness below 2 nm. Following the laser process, these ripples persist, accompanied by a reduction in the roughness to about 1 nm. Subsequent thermal annealing does not significantly alter the surface morphology. The SiGe film displays similar ripples, albeit less pronounced, resulting in a lower RMS roughness of approximately 1 nm. After the PLM treatment, the ripples become denser and the roughness slightly decreases. After thermal annealing, ripples comparable to those observed in GeA appear, with an overall roughness remaining low and not exceeding 0.5 nm. The as-grown GeB sample shows no ripple formation but instead presents several islands; however, the surface roughness remains around 1 nm. After PLM, these islands disappear, reducing the RMS roughness to about 0.5 nm. Finally, thermal annealing leads to a deterioration of surface morphology, increasing roughness to approximately 1.7 nm. In summary, under all examined conditions, the surface morphology undergoes only minor changes due to laser and thermal treatments, and the RMS roughness remains consistently low, suggesting that these samples are suitable substrates for subsequent epitaxial re-growth.

### Optical characterization

**Fig. 3:**
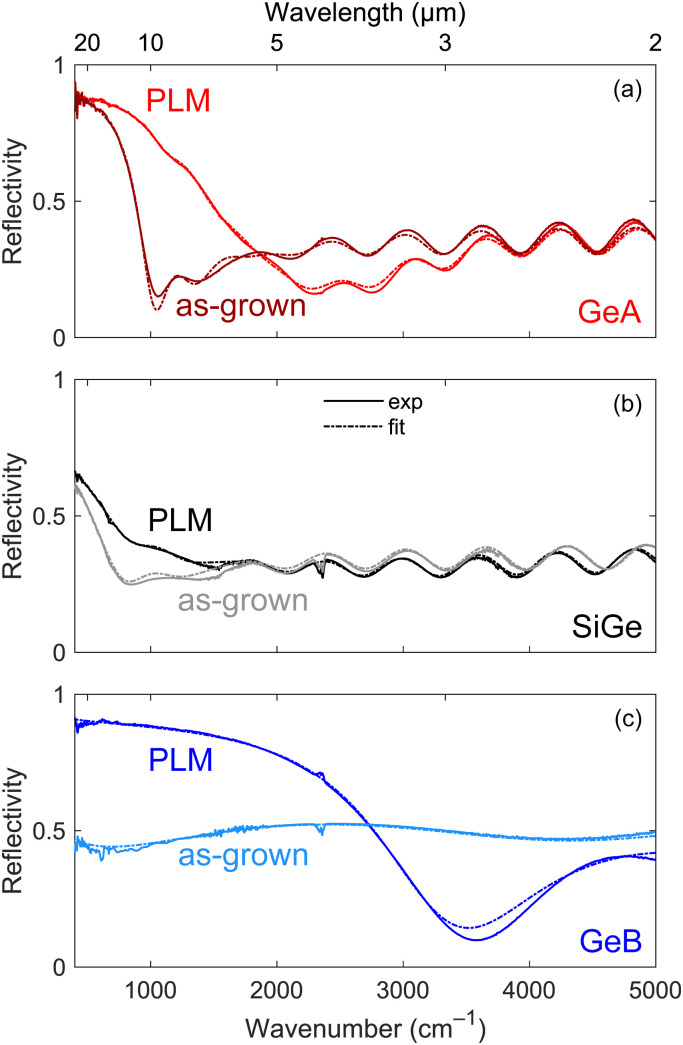
FTIR reflectivity spectra of (a) GeA, (b) SiGe and (c) GeB before and after the PLM process together with the best fitting curves obtained within the multilayer model.

The FTIR reflectivity spectra before and after the PLM process are presented in Fig. 3(a), (b) and (c) for GeA, SiGe and GeB, respectively. The reflectivity of as-grown GeA features a plasma edge around 1000 cm^−1^ (10 μm wavelength) followed by fringes determined by the interference due to the finite thickness of the film. After PLM, the plasma edge blue-shifts to around 2000 cm^−1^ (5 μm wavelength) and gets smoother, while the fringes appearing in the transparency region of the material basically remain unaltered. The as-grown SiGe sample features a plasma edge at around 800 cm^−1^ (12.5 μm wavelength). Given a comparable electron density to that of GeA, the observed red-shift can be mainly attributed to the larger effective mass. After the PLM, an increase of the reflectivity up to around 1500 cm^−1^ is observed, but it is not possible to clearly identify a plasma edge as the thickness of the hyper-doped region in the SiGe sample is reduced to approximately 180 nm which is lower than the skin depth. Finally, the as-grown GeB film does not show any clear plasma edge in the investigated spectral region due to the low carrier density, but after PLM, the huge increase in the active carrier concentration pushes the plasma edge to around 3300 cm^−1^ (3 μm wavelength), with a reflectivity well above 0.5 for most of the MIR spectral range, *i.e.* for wavelengths longer than 4 μm. Moreover, the period of the interference fringes is approximately four times larger with respect to GeA and SiGe because of the film being four times thinner.

The quantitative analysis of the spectra was carried out considering for each layer a Drude-like dielectric function, *i.e. ε*(*ω*) = *ε*_∞_ − *ω*_p_^2^/(*ω*^2^ + *iγω*) with *ε*_∞_, *ω*_p_ and *γ* being the high-frequency permittivity, the plasma frequency and the damping rates or losses, respectively. In order to limit the number of fitting parameters, the value of *ε*_∞_ was kept fixed. For both germanium samples a value of 16.2 was used, which already accounts for the tails of interband transitions occurring at higher energies.^33^ Similarly, the dielectric constant of SiGe alloys was computed as the weighted average of the high-frequency permittivities of pure germanium and silicon finding 15.5 for Si_0.15_Ge_0.85_. The values of the plasma frequency and of the losses retrieved by fitting the experimental spectra of Fig. 3 are reported in Table 2.

**Table 2:** Plasma frequency, electron density and losses retrieved from the FTIR spectra shown in Fig. 4. For comparison, the carrier concentration retrieved from electrical VdP–Hall characterization is also reported

Sample	As-grown				PLM			
	*ω*_p_ [cm^−1^]	*γ* [cm^−1^]	*n* [cm^−3^]	*n*_H_ [cm^−3^]	*ω*_p_ [cm^−1^]	*γ* [cm^−1^]	*n* [cm^−3^]	*n*_H_ [cm^−3^]
GeA	3985	215	2.1 × 10^19^	2.0 × 10^19^	6929	333	6.4 × 10^19^	6.1 × 10^19^
SiGe	2934	494	2.1 × 10^19^	1.1 × 10^19^	4806	749	5.7 × 10^19^	4.6 × 10^19^
GeB	—	—	—	3.4 × 10^18^	12 229	672	2.3 × 10^20^	2.6 × 10^20^

According to eqn (1), to extract the carrier concentration from the plasma frequency, the knowledge of the effective mass is needed. In germanium, the electron conductivity effective mass is expected to vary with the carrier concentration due to the non-parabolic dispersion of the conduction band in proximity of the *L* minima: however, as long as the electron concentration is lower than 10^20^ cm^−3^, the effective mass can be reasonably approximated to 0.12*m*_e_; when, instead, the carrier density gets higher than 10^20^ cm^−3^, the effective mass increases and approaches 0.14*m*_e_.^34^ Since experimental values for the effective mass of silicon–germanium alloys are, to the best of our knowledge, not available, a linear interpolation is usually performed using the known conductivity effective masses of silicon and germanium: for a Ge fraction *x* < 0.85, the conduction band minima are located at *Δ* and the electrons effective mass is approximately 
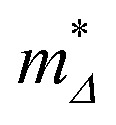
, *i.e.* 0.26*m*_e_, while for *x* > 0.85 the minima are at *L* and the effective mass is approximately 
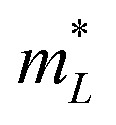
, *i.e.* 0.12*m*_e_. At *x* ≃ 0.85 the alloy character changes from Si- to Ge-like, meaning that the *Δ* and *L* valleys are almost degenerate, thus both populated, and a mere weighted average based on the Ge content cannot satisfactorily account for this effect. If two distinct electron populations coexist, the overall conductivity must be calculated considering both contributions. Assuming that all electrons show similar scattering times and that the occupancy of each valley only depends on the corresponding effective density of states *N*, the effective mass of the system can be calculated as2
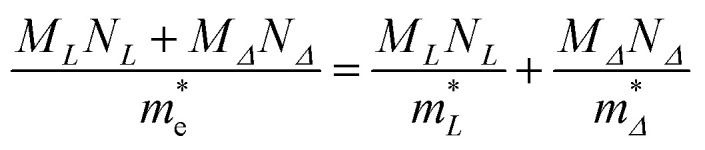
where *M*_*L*_ = 4 and *M*_*Δ*_ = 6 are the valley degeneracy while *N*_*L*_ = 1 × 10^19^ cm^−3^ and *N*_*Δ*_ = 3.2 × 10^19^ cm^−3^ are the effective density of states of the two valleys in pure germanium and silicon, respectively. From eqn (2), the electron effective mass 0.22*m*_e_ in Si_0.15_Ge_0.85_ is obtained. Applying eqn (1) with the proper effective mass, the electron density can thus be deduced obtaining values around 2 × 10^19^ cm^−3^ for the as-grown GeA and SiGe films; after PLM, instead, the electron density increases up to around 6 × 10^19^ cm^−3^. Eventually, in GeB, a carrier density in the hyper-doped layer exceeding 2.5 × 10^20^ cm^−3^ was found.

**Fig. 4:**
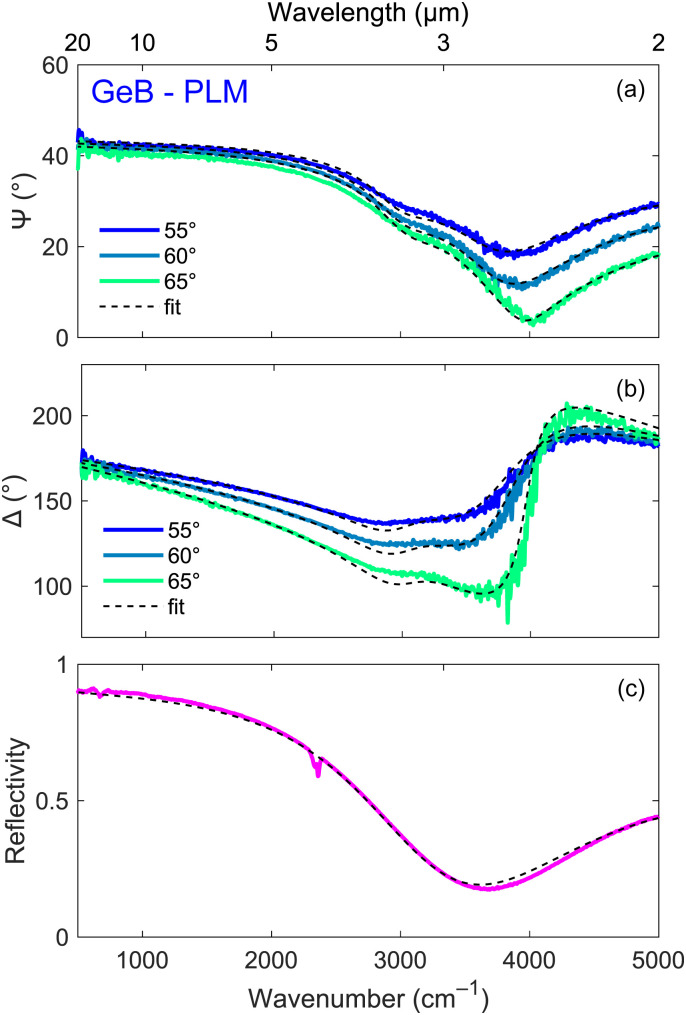
Ellipsometric angles (a) *Ψ* and (b) *Δ* and (c) FTIR reflectivity spectrum of GeB after the PLM process. The corresponding best fitting curves (dashed lines) are also shown.

In addition to the FTIR characterization, IRSE measurements were performed on selected samples to validate the multilayer model. Fig. 4 shows the ellipsometric angles *Ψ* and *Δ*, together with the FTIR reflectivity of PLM-activated GeB. The experimental data were well reproduced using a multilayer model that includes a frequency-dependent damping rate *γ*(*ω*) in the Drude function for the hyper-doped Ge layer.^33^ However, the extracted electron density differs by less than 1% from the one obtained using a simpler model with constant *γ*, which was therefore employed throughout this work to reliably estimate the carrier concentration from the FTIR spectra.

The electron densities obtained in this way are reported in Table 2 together with the ones retrieved by electrical VdP–Hall measurements. The accordance is excellent both in GeA and GeB with the small differences being ascribed to the fact that they refer to two nominally identical still different samples. The accordance worsens for SiGe possibly because of the rough estimates adopted for the electron effective mass and the Hall scattering factor.

### Isothermal treatments

To investigate the thermal stability of the hyper-doped Ge and SiGe films, thermal treatments were performed under different annealing conditions and the carrier concentration after each process was retrieved from the FTIR reflectivity spectra.

**Fig. 5:**
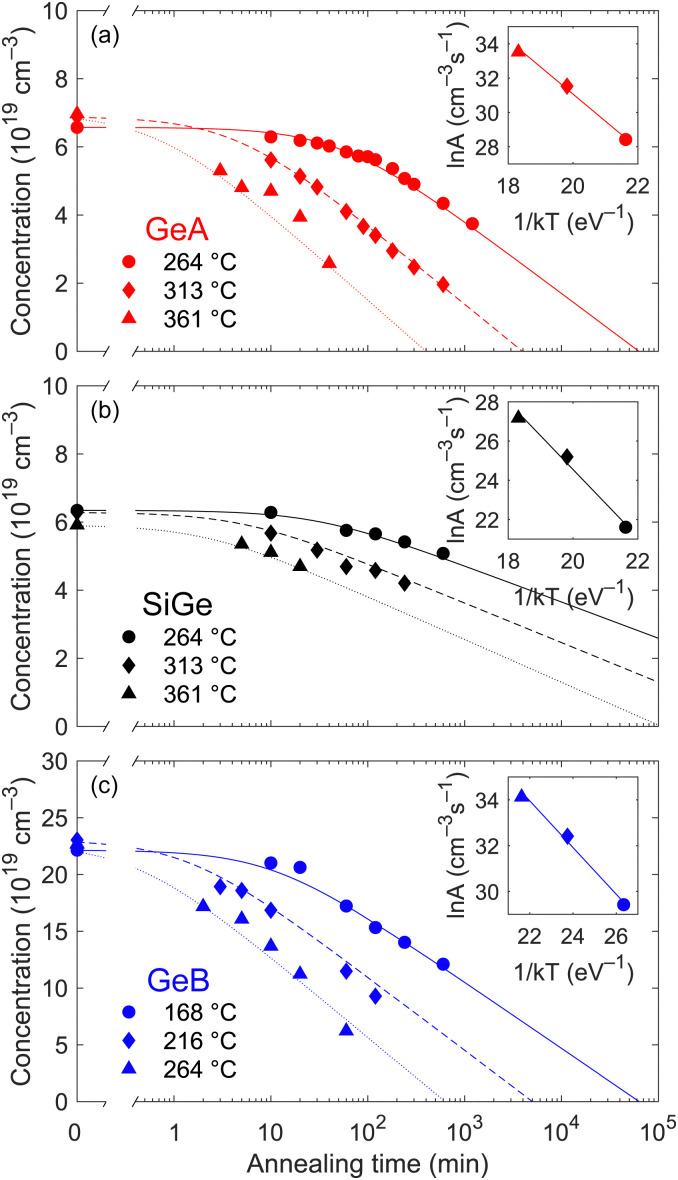
Evolution of the electron density in the hyper-doped layer at different temperatures as a function of the annealing time for (a) GeA, (b) SiGe and (c) GeB. The insets show the Arrhenius plots employed to retrieve *A*_0_ and *E*_a_.

Assuming the duration of the heating and cooling ramps to be negligible, each isothermal curve was obtained by repeating thermal treatments at a fixed temperature on the same sample for different times, effectively mimicking the effect of a single uninterrupted treatment with the same total duration. Isothermal curves were acquired at 264, 313 and 361 °C for GeA and SiGe and at 168, 216 and 264 °C for GeB. The lower annealing temperatures for GeB were selected following preliminary treatments which revealed a more rapid deactivation dynamics with respect to the other samples. The evolution of the electron density during these processes is reported in Fig. 5 as a function of the cumulative annealing time.

The deactivation dynamics can be analysed quantitively in the framework of the model introduced by Nobili *et al.*^35,36^ to study the thermally-induced deactivation of arsenic dopants in silicon. According to this model, the deactivation process can be approximated by the rate equation3
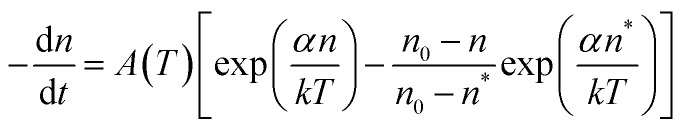
with *A*(*T*) = *A*_0_ exp(−*E*_a_/*kT*), where *A*_0_ and *α* are temperature-independent parameters, *E*_a_ is the activation energy, and *n*_0_ and n* are the initial and equilibrium electron densities, respectively. This latter, in particular, changes with temperature and is determined by the equilibrium between the formation and dissolution of clusters and complexes. When *n* ≫ n*, *i.e.* far from saturation, the second term in square brackets can be neglected and eqn (3) can be solved analytically giving4
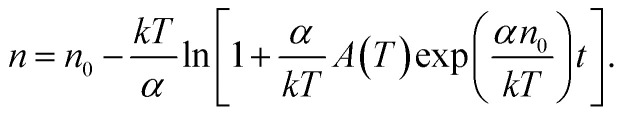


For long annealing treatments, the carrier concentration decreases linearly with the logarithm of the annealing time. When instead *n* starts approaching n*, the second term in eqn (3) cannot be neglected anymore and the rate equation can only be solved numerically; however, as it can be seen in Fig. 5, no deviations from the linear trend of *n* as a function of ln *t* were observed for the considered annealing conditions, meaning that this regime was never reached. The isothermal curves in Fig. 5 were consequently fitted according to eqn (4) eventually finding a good agreement with the experimental data and allowing one to retrieve *α* and *A*(*T*). Exploiting the linear relation between ln *A* and 1/*T*, the values of *A*(*T*) were used to extract *A*_0_ and *E*_a_. The corresponding Arrhenius plots referring to each sample are shown in the insets of Fig. 5. The parameters retrieved from the fitting of the isothermal curves are reported in Table 3.

**Table 3:** Parameters extracted from the fitting of the isothermal curves shown in Fig. 6 according to eqn (4)

Sample	*α* [eV cm^3^]	*A*_0_ [cm^−3^ s^−1^]	*E*_a_ [eV]
GeA	5.0 × 10^−21^	1.67 × 10^27^	1.58
SiGe	1.0 × 10^−20^	4.09 × 10^25^	1.72
GeB	1.5 × 10^−21^	3.13 × 10^24^	1.02

**Fig. 6:**
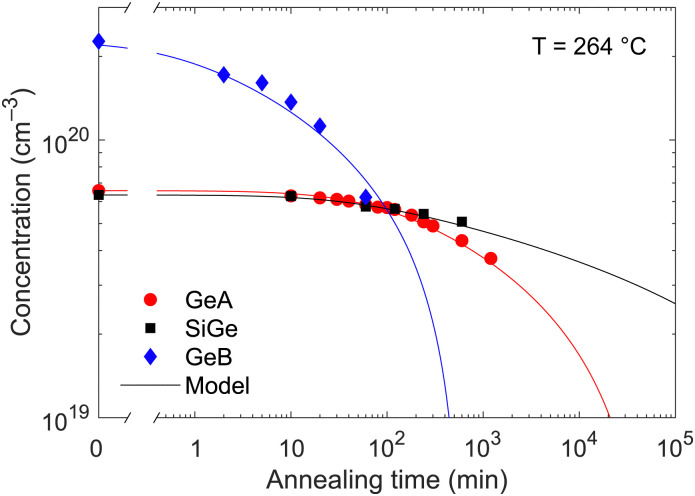
Evolution of the electron density in the hyper-doped layer at 264 °C for the three samples, together with the carrier density calculated according to eqn (4) by using the parameters reported in Table 3.

Although the complexity of eqn (4) prevents a full disentanglement of the contribution of each parameter, some general observations can still be made. At a given temperature, the slope of the isothermal curves at long annealing times is primarily governed by *α*, whereas the activation onset is mainly influenced by the combined effect of *A*_0_ and *E*_a_. Among the studied samples, SiGe, which is characterized by the highest activation energy *E*_a_ and *α*, exhibits the greatest resilience to thermal deactivation, only showing minimal reduction in active carrier density even after several hours of annealing at the considered temperatures. In contrast, GeB already starts deactivating after only a few minutes, due to its low activation energy, and the active carrier density rapidly drops, mainly because of the value of *α* which is the lowest among those of the considered samples. Finally, GeA exhibits an intermediate behaviour. To better illustrate these results, Fig. 6 presents a comparison among the isothermal curves of the three samples measured at 264 °C.

These distinct behaviours influence the suitability of each material for specific applications. For example, the formation of low-resistivity ohmic contacts typically requires annealing at temperatures of at least 300 °C for a few minutes.^21^ Under these conditions, all investigated materials remain viable; however, at higher annealing temperatures, GeA and SiGe are preferable to GeB due to their superior thermal stability. Similarly, while typical consumer electronics operate below 100 °C, where deactivation effects are minimal in all materials, high-power applications may involve operating temperatures exceeding 150 °C, where GeB is prone to significant degradation. In such cases, GeA or SiGe are preferable due to their enhanced thermal stability. For MIR optoelectronic applications, however, the exceptionally high carrier concentration achievable in GeB remains critical. In this case, the most thermally-demanding post-PLM step is the epitaxial growth of additional layers on top of the hyper-doped material which must therefore be carefully optimized. Low-temperature epitaxial growth using techniques such as LEPECVD can be performed at temperatures as low as 250 °C for durations of a few tens of minutes. Although notable dopant deactivation is expected in GeB under these conditions, carrier concentrations exceeding 10^20^ cm^−3^ can still be maintained.

It is worth noting the strikingly different sets of parameters obtained for GeA and GeB. Indeed, as both samples are made of germanium, one would expect the parameters to be similar. However, the pronounced differences suggest that the peculiar growth conditions, likely leading to an increased vacancy concentration, or the remarkably high density of incorporated phosphorus atoms can significantly alter the deactivation dynamics, despite the material itself being the same. Positron annihilation lifetime spectroscopy will be performed aiming at better identifying the origin of these differences and at shedding light on the microscopic mechanism responsible for the thermally-induced deactivation.

### Isochronal thermal treatments

**Fig. 7:**
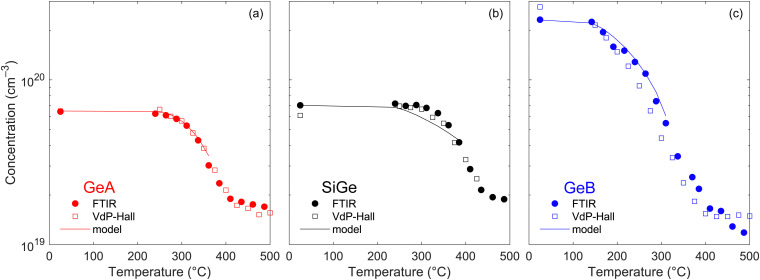
Evolution of the electron density during subsequent isochronal annealing treatments performed at gradually increasing temperature for (a) GeA, (b) SiGe and (c) GeB. The concentration was extracted from the FTIR reflectivity spectra (solid symbols) and from conventional VdP–Hall electrical measurements (open symbols). The simulated curves calculated with the parameters of Table 3 are also shown (solid lines).

To gain further insights into the deactivation dynamics, isochronal thermal treatments, lasting 10 minutes each, were performed on the three materials after PLM, with the annealing temperature progressively being increased up to 500 °C and starting from 220 °C for GeA and SiGe and from 140 °C for GeB. The active carrier density was systematically characterized both by electrical VdP–Hall and FTIR reflectivity measurements. The results are shown in Fig. 7 where only minimal differences can be observed between the carrier densities measured by the two techniques, confirming that FTIR spectroscopy is a fast and reliable method for determining this quantity in hyper-doped samples with a high-enough doping.

Examining the deactivation dynamics, GeA remains stable up to around 250 °C, after which the carrier density sharply decreases, exhibiting an inflection point near 360 °C and approaching values close to those of the as-grown material. SiGe instead demonstrates greater thermal stability, with deactivation initiating around 300 °C and progressing more gradually, characterized by an inflection point at 380 °C. In contrast, GeB shows an early onset of deactivation beginning at 150 °C, followed by a steady decrease in the active carrier density and an inflection point at approximately 310 °C. Notably, the active carrier density in GeB does not recover the as-grown value but saturates at around 2 × 10^19^ cm^−3^, hence suggesting that the as-grown film exhibit an out-of-equilibrium state which does not correspond to the thermodynamically stable configuration.

Nobili's model can also be applied to quantitatively analyze the isochronal treatments. However, it is important to notice that the model fits the data only up to a certain threshold temperature, above which the de-clustering term can be safely neglected. Assuming that this threshold corresponds to the inflection point of the curves, eqn (4) is applicable up to 360, 385, and 310 °C for GeA, SiGe, and GeB, respectively. Using the parameters reported in Table 3, a nice agreement between the simulated and experimental curves was obtained up to these temperatures for GeA and GeB while the model slightly underestimates the active carrier density in the SiGe samples.

## Conclusions

This work presents a detailed investigation into the deactivation dynamics of hyper-doped n-type Ge and Si_0.15_Ge_0.85_ epilayers grown on silicon substrates and processed by PLM with the aim of evaluating their suitability for MIR optoelectronic and plasmonic applications. Our results demonstrate that PLM is a highly effective technique for enhancing dopant activation, enabling electron densities exceeding 5 × 10^19^ cm^−3^ not only in Ge but also in SiGe films.

Among the investigated materials, Ge films grown under low-temperature and low-rate conditions stand out for achieving the highest active carrier concentration, up to 2.6 × 10^20^ cm^−3^, and exhibiting exceptional reflectivity over the MIR range. These characteristics make these materials particularly promising for MIR mirrors and other optical components where high reflectivity and optical conductivity are critical. However, their rapid deactivation at moderate annealing temperatures constrains their integration into thermally demanding fabrication steps or multilayer stacks requiring post-PLM growth. In contrast, Ge films grown under standard conditions achieve lower active carrier densities due to reduced P incorporation, but benefit from improved thermal stability. This may offer a practical compromise for devices which require both optical and electrical functionality without extreme processing constraints. Finally, Si_0.15_Ge_0.85_ shows the highest thermal robustness, retaining carrier activation even after prolonged annealing. However, its higher effective mass compared to that of Ge significantly lowers its reflectivity, making it less suitable for optical components and better suited to electrically driven applications or integration schemes where thermal compatibility is prioritized over optical performance.

More broadly, our findings highlight the importance of selecting hyper-doped material platforms not solely based on peak carrier density or thermal stability, but on the specific functional priorities of the target application, *e.g.* MIR reflectivity, doping uniformity, or compatibility with subsequent processing. Furthermore, the preservation of smooth surface morphology across all samples, especially after PLM, suggests that these layers may serve as suitable platforms for subsequent epitaxial regrowth, after careful optimization of the thermal budget, enabling integration into more complex optoelectronic and photonic devices.

## Author contributions

M. Faverzani: methodology, formal analysis, investigation, data curation, writing – original draft, writing – review & editing, visualization. G. M. Spataro: methodology, formal analysis, investigation, data curation, writing – review & editing. D. Impelluso: methodology, investigation, writing – review & editing. S. Calcaterra: methodology, investigation, writing – review & editing. E. Di Russo: methodology, investigation, writing – review & editing. M. Magnozzi: investigation, writing – review & editing. F. Bisio: investigation, writing – review & editing. M. Canepa: investigation, writing – review & editing. P. Biagioni: methodology, writing – review & editing, supervision. G. Isella: methodology, writing – review & editing, supervision. E. Napolitani: conceptualization, methodology, writing – review & editing, supervision, project administration, funding acquisition. J. Frigerio: conceptualization, methodology, writing – review & editing, supervision, project administration, funding acquisition.

## Conflicts of interest

There are no conflicts to declare.

## Data Availability

The data that support this study are openly available in Zenodo at https://doi.org/10.5281/zenodo.15698678.
